# Assessment of Patients’ Quality-of-Life Post-Thyroidectomy

**DOI:** 10.7759/cureus.52744

**Published:** 2024-01-22

**Authors:** Sahar M Alnefaie, Muath S Alotaibi, Abdullah E Alsobaie, Wafi B Alotaibi, Mohammad I Alwuthaynani, Ahmed R Alotibi, Fahad A Alayyaf, Rasan F Almnjwami

**Affiliations:** 1 Department of Surgery, College of Medicine, Taif University, Taif, SAU

**Keywords:** saudi arabia, middle east, thyroid cancer, quality of life, thyroidectomy, survivorship

## Abstract

Introduction: The assessment of quality of life (QOL) after thyroidectomy has been a focus of medical research, aiming to understand its impact on patients' overall well-being and functional status. Studies have examined the physical, psychological, and social dimensions of QOL, providing valuable insights into the outcomes of thyroidectomy and improving patient care. The aim of the study is to evaluate the long-term impact of different aspects of patients' quality of life post-thyroidectomy.

Methods: This cross-sectional study was conducted in Taif City and included 100 participants post-thyroidectomy. Data were collected from medical records and telephone interviews using the modified Arabic version of the EROTC QLQ-H&N43 questionnaire. The collected data were analyzed using R software version 4.2.2.

Results: The majority of the 100 participants were female (76%), and most were over 50 years old (38%). The types of surgery performed included hemithyroidectomy (36%), subtotal thyroidectomy (6%), and total thyroidectomy (58%). Participants reported difficulties related to their senses, body satisfaction, sexual satisfaction, eating, speaking, and social interactions. Pain in the mouth and jaw, as well as swallowing difficulties, showed variations among the surgery groups. Other symptoms, such as tooth problems, dry mouth, and swelling in the neck, did not differ significantly among the groups. Numbness in the hands or feet was associated with a subtotal or total thyroidectomy. Difficulties with enjoying meals, eating in front of others, and communication issues showed variations among the surgery groups.

Conclusion: This study provided insights into the QOL of thyroidectomy survivors in Taif City. Participants reported challenges related to sensory, body, and sexual issues, as well as difficulties with eating, speaking, and social interactions. The findings highlight the need for interventions and support to address these challenges and optimize the QOL of thyroidectomy patients. Furthermore, research is warranted to explore specific factors contributing to these difficulties and to develop targeted interventions for better patient outcomes.

## Introduction

Thyroid carcinoma (TC) is the endocrine system's most often diagnosed cancer and one of the ten most common cancers in 2020. Incidence rates in females are three times greater globally than in males (10.1 versus 3.1 per 100,000). In 2020, there were 586,000 new instances of TC worldwide; nevertheless, the incidence of cancer-related mortality remained very low, with an anticipated 44,000 deaths in both genders combined (0.5 per 100,000 in women and 0.3 per 100,000 in men) [1]. The Surveillance, Epidemiology, and End Results (SEER) 2011-2017 data indicates that 98.3% of TC patients had a five-year relative survival rate. 65.9% of TC cases are caused by localized tumors, which have a 99.9% five-year relative survival rate, which is considered excellent [2]. For Saudi women, thyroid cancer came in second, and for Saudi men, it came in tenth. In 2020, 1044 cases of thyroid cancer were reported among Saudi nationals, making up 7.4% of all newly diagnosed cancer cases. There were 228 (21.8%) males and 816 (78.2%) females affected by thyroid cancer, for a female-to-male ratio of 358 to 100 [3].

The assessment of quality of life (QOL) post-thyroidectomy has gained significant attention in the medical community. Multiple studies have delved into the long-term consequences of thyroidectomy, aiming to evaluate the impact on patients' overall well-being and functional status [4,5]. By examining numerous aspects, such as the physical, psychological, and social dimensions of QOL, researchers have provided valuable insights into the outcomes of thyroidectomy, hence contributing to improving patient care and enhancing their postoperative experiences.

Examinations of the physical dimension of QOL have indicated that most individuals who have undergone thyroidectomy in Taif achieve satisfactory outcomes in terms of overall physical health. Research has focused on various aspects, such as voice quality, swallowing function, and scar appearance. Findings suggest that most patients report no significant changes in their voice or swallowing after thyroidectomy, providing reassurance and reducing concerns regarding potential negative effects. Additionally, studies have investigated the cosmetic outcomes of surgery, highlighting the importance of surgeon experience and techniques in minimizing scar visibility, ultimately leading to greater patient satisfaction [6].

Regarding the psychological dimension of QOL, several studies have emphasized patients' emotional well-being post-thyroidectomy. Findings have demonstrated that, while many individuals experience temporary emotional disturbances related to the surgery, long-term psychological outcomes generally improve over time. In fact, the majority of patients report higher levels of self-esteem and body image post-thyroidectomy, as the removal of a diseased or abnormal thyroid gland can alleviate anxiety and distress associated with the underlying condition. However, additional research is needed to explore the specific factors contributing to these positive psychological outcomes and to identify potential interventions for those experiencing psychological distress following thyroidectomy [7,8].

The social dimension of QOL post-thyroidectomy has also been explored, aiming to understand the impact of the surgery on patients' social interactions and overall social well-being. Studies in this area have indicated that most individuals do not perceive any negative consequences on their social lives after thyroidectomy. However, some patients may experience challenges adjusting to changes in their appearance or vocal function, which can potentially affect their confidence and social interactions. Further investigation into the specific areas causing social difficulties, along with interventions to support patients in overcoming these challenges, would be beneficial for optimizing the social dimension of QOL post-thyroidectomy [9].

Certain individuals may require lifelong assistance in adjusting to thyroid hormone replacement therapy following a thyroidectomy [7]. Despite an increasing body of evidence in the medical literature showing these patients' unmet needs [7,10], physicians still frequently underestimate the difficulties these patients encounter [11]. Moreover, only a limited number of studies [12,13] have investigated the long-term impact on quality of life (QOL). To bridge the gap between patients' needs and the services provided and to close the gap that exists between the services offered and the needs of the patients [14], it is crucial to develop an effective postoperative care program to enhance the long-term QOL of thyroid cancer survivors. This study addresses this knowledge gap by evaluating the long-term QOL (5-15 years post-thyroidectomy) of thyroid tumor survivors and identifying the factors significantly associated with a negative impact.

## Materials and methods

This study employed a cross-sectional design to examine the quality of life among thyroidectomy patients in Taif City. The study population consisted of adults aged 12 years and older who were followed for 5 to 15 years post-thyroidectomy due to either benign or malignant causes. Individuals who had undergone surgery less than five years ago or more than 15 years ago, those younger than 12, and participants residing outside of Taif City were excluded from the study.

Al Hada Armed Forces Hospital, Prince Mansour Military Hospital, and Prince Sultan Military Hospital were the three government hospitals from which the data was gathered. From, which we identified 537 patients and contacted them. Only 100 individuals were included in the study; the remaining patients were not surgically treated, were misdiagnosed, died, or refused to be part of the research. From the medical records system, contact details and basic demographic information (gender, age, socioeconomic status, precise diagnosis, number of years post-surgery) were retrospectively gathered and confirmed during the phone survey. 

The Arabic version of the European Organization for Research and Treatment of Cancer's head and neck cancer-specific quality of life questionnaire (EROTC QLQ) [6] - H&N43 was filled out by the participants over the phone. The questionnaire has been modified using yes or no questions.

Data analysis

The collected data underwent cleaning and organization in an Excel spreadsheet before being imported into R software version 4.2.2. Descriptive statistics were used to analyze continuous data using the mean and standard deviation, while categorical data were summarized with frequencies and percentages. A Fisher exact test was used to assess the relationship between the type of thyroidectomy surgery and respondent pain and swallowing difficulties. It was also used to draw an association between the type of thyroidectomy surgery and trouble with social eating and social contact among the respondents. 

Ethical considerations

Three government hospitals, Prince Mansour Military Hospital, Prince Sultan Military Hospital, and Al Hada Armed Forces Hospital, gave their ethical approval for this study (Registration No. H-02-T-078). Informed consent was obtained from participants after a clear explanation of the study's objectives. Participants were assured of their right to withdraw at any point, and the anonymity and confidentiality of their responses were rigorously maintained throughout the study.

## Results

In this study of 100 participants, the demographic characteristics are described in Table 1.

**Table 1:** Demographic characteristics of the study participants 1: n (%)

Characteristic	N = 100^1^
Gender	
Female	76 (76%)
Male	24 (24%)
Age	
Less than 40 years	25 (25%)
40-50 years	37 (37%)
More than 50 years	38 (38%)
Marital status	
Single	7 (7.0%)
Married	72 (72%)
Divorced	6 (6.0%)
Widowed	15 (15%)
Educational level	
Primary school	31 (31%)
Middle school	12 (12%)
High school	20 (20%)
Bachelor’s	30 (30%)
Diploma	2 (2.0%)
Post-graduate	5 (5.0%)
Occupation	
Government employee	19 (19%)
Private sector employee	6 (6.0%)
Private job	4 (4.0%)
Student	5 (5.0%)
Health care provider	2 (2.0%)
I’m not working right now	64 (64%)
Type of surgery	
Hemithyroidectomy	36 (36%)
Subtotal thyroidectomy	6 (6.0%)
Total thyroidectomy	58 (58%)
In which year did you have the surgery?	
2005-2008	13 (13%)
2009-2012	16 (16%)
2013 and later	71 (71%)

Most participants were female (76%) and over 50 years old (38%). Most participants were married (72%), had primary school education (31%), and were not currently working (64%). The types of surgery performed were hemithyroidectomy (36%), subtotal thyroidectomy (6%), and total thyroidectomy (58%). 

A notable proportion reported experiencing various pains and swallowing difficulties. Specifically, 18% reported pain in the mouth, 16% in the jaw, and 46% in the throat. Swallowing problems were reported by 22% of participants, with 37% experiencing difficulties swallowing solid food. Additionally, 48% reported problems with their teeth, 27% had issues due to tooth loss, and 38% felt numbness in their hands or feet, as depicted in Table 2. 

**Table 2:** Respondent pain and swallowing difficulties 1: n (%)

Characteristic	N = 100^1^
Have you had pain in your mouth?	
No	82 (82%)
Yes	18 (18%)
Have you had pain in your jaw?	
No	84 (84%)
Yes	16 (16%)
Have you had pain in your throat?	
No	54 (54%)
Yes	46 (46%)
Have you had problems swallowing?	
No	78 (78%)
Yes	22 (22%)
Have you had problems swallowing liquids?	
No	79 (79%)
Yes	21 (21%)
Have you had problems swallowing pureed food?	
No	79 (79%)
Yes	21 (21%)
Have you had problems swallowing solid food?	
No	63 (63%)
Yes	37 (37%)
Have you choked when swallowing?	
No	82 (82%)
Yes	18 (18%)
Have you had problems opening your mouth wide?	
No	78 (78%)
Yes	22 (22%)
Have you had problems with your teeth?	
No	52 (52%)
Yes	48 (48%)
Have you had problems because of losing some teeth?	
No	73 (73%)
Yes	27 (27%)
Have you had a dry mouth?	
No	71 (71%)
Yes	29 (29%)
Have you had sticky saliva?	
No	80 (80%)
Yes	20 (20%)
Have you had swelling in your neck?	
No	71 (72%)
Yes	28 (28%)
Missing	1
Have you had pain in your shoulder?	
No	72 (72%)
Yes	28 (28%)
Have you had problems raising your arm or moving it sideways?	
No	77 (77%)
Yes	23 (23%)
Have you felt numbness in your hands or feet?	
No	62 (62%)
Yes	38 (38%)

Regarding the problems related to senses, body satisfaction, and sexual satisfaction, 19% felt dissatisfied with their bodies and experienced weight loss. Among those with weight loss, six (32%) expressed worry about it. Some participants experienced problems with their sense of smell (12%) and taste (13%). Additionally, 20% reported decreased interest in sex, while 22% felt less sexual enjoyment. Skin problems were reported by 38% of participants, including rash (15%) and skin color change (18%). Concerns about test results and future health were expressed by 57% and 63% of participants, respectively. Difficulties in injury healing were reported by 16% of participants, as depicted in Table 3.

**Table 3:** Senses problems, body, and sexual satisfaction among the respondent 1: n (%)

Characteristic	N = 100^1^
Have you felt dissatisfied with your body?	
No	81 (81%)
Yes	19 (19%)
Have you felt your weight become lower?	
No	81 (81%)
Yes	19 (19%)
If your weight becomes low, does that worry you?	
No	13 (68%)
Yes	6 (32%)
Missing	81
Have you had problems with your sense of smell?	
No	88 (88%)
Yes	12 (12%)
Have you had problems with your sense of taste?	
No	87 (87%)
Yes	13 (13%)
Have you felt less interest in sex?	
No	80 (80%)
Yes	20 (20%)
Have you felt less sexual enjoyment?	
No	78 (78%)
Yes	22 (22%)
Have you had skin problems?	
No	62 (62%)
Yes	38 (38%)
Have you had a rash?	
No	85 (85%)
Yes	15 (15%)
Have you had a skin color change?	
No	82 (82%)
Yes	18 (18%)
Have you felt worried because of the test results?	
No	43 (43%)
Yes	57 (57%)
Are you worried about your health in the future?	
No	37 (37%)
Yes	63 (63%)
Have you had difficult injury healing?	
No	84 (84%)
Yes	16 (16%)

A significant proportion reported difficulties in eating, speaking, and social interactions. Notably, 25% experienced problems enjoying meals, 10% had difficulties eating in front of their family, and 17% faced challenges eating in front of others. Communication issues were also prevalent, with 19% reporting problems talking to others, talking on the telephone, and speaking in a noisy environment. Additionally, 20% had difficulties speaking clearly, and 29% experienced problems with coughing. Social participation was affected by 10% of participants who reported difficulties going out in public, as depicted in Table 4.

**Table 4:** Trouble with social eating and social contact among the respondents 1: n (%)

Characteristic	N = 100^1^
Have you had problems enjoying your meals?	
No	75 (75%)
Yes	25 (25%)
Have you had problems eating in front of your family?	
No	90 (90%)
Yes	10 (10%)
Have you had problems eating in front of other people?	
No	83 (83%)
Yes	17 (17%)
Have you had problems talking to other people?	
No	81 (81%)
Yes	19 (19%)
Have you had problems talking on the telephone?	
No	81 (81%)
Yes	19 (19%)
Have you had problems talking in a noisy environment?	
No	75 (75%)
Yes	25 (25%)
Have you had problems speaking clearly?	
No	80 (80%)
Yes	20 (20%)
Have you had problems with coughing?	
No	71 (71%)
Yes	29 (29%)
Have you had problems going out in public?	
No	90 (90%)
Yes	10 (10%)

A significant association was observed between the type of surgery and numbness in the hands or feet (p = 0.041). A higher percentage of individuals who underwent subtotal thyroidectomy (3, 50%) or total thyroidectomy (27, 47%) reported experiencing numbness in their hands or feet compared to those who had a hemithyroidectomy (8, 22%). 

A higher percentage of respondents reported experiencing pain in the mouth in the subtotal thyroidectomy group (33%) compared to the other two groups. A higher proportion of individuals in the subtotal thyroidectomy group reported experiencing jaw pain (33%) compared to the other groups (11% and 17% for hemithyroidectomy and total thyroidectomy, respectively). Respondents in the total thyroidectomy group reported the highest percentage of throat pain (52%), followed by the subtotal thyroidectomy group (50%) and the hemithyroidectomy group (36%). 

It is noteworthy that the subtotal thyroidectomy group had the highest percentage of individuals reporting problems with swallowing three (50%), followed by the total thyroidectomy group 12 (21%), and the hemithyroidectomy group seven (19%). However, the subtotal thyroidectomy group had the highest percentage of respondents reporting problems with teeth and losing teeth (17% and 22%, respectively), as depicted in Table 5.

**Table 5:** Association between type of surgery and respondent pain and swallowing difficulties 1: n (%), 2: Fisher's exact test

	Type of surgery			
Characteristic	Hemithyroidectomy, N = 36^1^	Subtotal thyroidectomy, N = 6^1^	Total thyroidectomy, N = 58^1^	p-value^2^
Have you had pain in your mouth?				0.5
No	30 (83%)	4 (67%)	48 (83%)	
Yes	6 (17%)	2 (33%)	10 (17%)	
Have you had pain in your jaw?				0.3
No	32 (89%)	4 (67%)	48 (83%)	
Yes	4 (11%)	2 (33%)	10 (17%)	
Have you had pain in your throat?				0.3
No	23 (64%)	3 (50%)	28 (48%)	
Yes	13 (36%)	3 (50%)	30 (52%)	
Have you had problems swallowing?				0.2
No	29 (81%)	3 (50%)	46 (79%)	
Yes	7 (19%)	3 (50%)	12 (21%)	
Have you had problems swallowing liquids?				0.6
No	30 (83%)	4 (67%)	45 (78%)	
Yes	6 (17%)	2 (33%)	13 (22%)	
Have you had problems swallowing pureed food?				0.6
No	30 (83%)	4 (67%)	45 (78%)	
Yes	6 (17%)	2 (33%)	13 (22%)	
Have you had problems swallowing solid food?				0.5
No	25 (69%)	3 (50%)	35 (60%)	
Yes	11 (31%)	3 (50%)	23 (40%)	
Have you choked when swallowing?				>0.9
No	29 (81%)	5 (83%)	48 (83%)	
Yes	7 (19%)	Yes (17%)	10 (17%)	
Have you had problems opening your mouth wide?				0.7
No	29 (81%)	4 (67%)	45 (78%)	
Yes	7 (19%)	2 (33%)	13 (22%)	
Have you had problems with your teeth?				0.2
No	19 (53%)	Yes (17%)	32 (55%)	
Yes	17 (47%)	5 (83%)	26 (45%)	
Have you had problems because of losing some teeth?				0.4
No	28 (78%)	3 (50%)	42 (72%)	
Yes	8 (22%)	3 (50%)	16 (28%)	
Have you had a dry mouth?				0.3
No	29 (81%)	4 (67%)	38 (66%)	
Yes	7 (19%)	2 (33%)	20 (34%)	
Have you had sticky saliva?				0.5
No	28 (78%)	4 (67%)	48 (83%)	
Yes	8 (22%)	2 (33%)	10 (17%)	
Have you had swelling in your neck?				0.7
No	24 (69%)	4 (67%)	43 (74%)	
Yes	11 (31%)	2 (33%)	15 (26%)	
Missing	Yes	No	No	
Have you had pain in your shoulder?				0.6
No	28 (78%)	4 (67%)	40 (69%)	
Yes	8 (22%)	2 (33%)	18 (31%)	
Have you had problems raising your arm or moving it sideways?				0.2
No	31 (86%)	4 (67%)	42 (72%)	
Yes	5 (14%)	2 (33%)	16 (28%)	
Have you felt numbness in your hands or feet?				0.041
No	28 (78%)	3 (50%)	31 (53%)	
Yes	8 (22%)	3 (50%)	27 (47%)	

A higher percentage of individuals who underwent a hemithyroidectomy reported experiencing difficulties in enjoying meals (8, 22%) compared to those who had a subtotal thyroidectomy (2, 33%) or a total thyroidectomy (15, 26%). 

A significant association (p = 0.046) was found between coughing and surgery type; individuals who underwent subtotal thyroidectomy reported more difficulties with coughing (4, 67%) compared to those who had a hemithyroidectomy (7, 19%) or a total thyroidectomy (18, 31%). Furthermore, no significant differences were found among the surgery groups in terms of problems going out in public (p = 0.9), enjoying meals (p = 0.7), eating in front of family (p = 0.4), or others (p = 0.8), and speaking problems (p > 0.05), as depicted in Table 6.

**Table 6:** Association between type of surgery and trouble with social eating and social contact among the respondents 1: n (%), 2: Fisher's exact test

	Type of surgery			
Characteristic	Hemithyroidectomy, N = 36^1^	Subtotal thyroidectomy, N = 6^1^	Total thyroidectomy, N = 58^1^	p-value^2^
Have you had problems enjoying your meals?				0.7
No	28 (78%)	4 (67%)	43 (74%)	
Yes	8 (22%)	2 (33%)	15 (26%)	
Have you had problems eating in front of your family?				0.4
No	34 (94%)	6 (100%)	50 (86%)	
Yes	2 (5.6%)	No (No%)	8 (14%)	
Have you had problems eating in front of other people?				0.8
No	30 (83%)	6 (100%)	47 (81%)	
Yes	6 (17%)	No (No%)	11 (19%)	
Have you had problems talking to other people?				0.2
No	32 (89%)	4 (67%)	45 (78%)	
Yes	4 (11%)	2 (33%)	13 (22%)	
Have you had problems talking on the telephone?				0.7
No	31 (86%)	5 (83%)	45 (78%)	
Yes	5 (14%)	Yes (17%)	13 (22%)	
Have you had problems talking in a noisy environment?				>0.9
No	27 (75%)	5 (83%)	43 (74%)	
Yes	9 (25%)	Yes (17%)	15 (26%)	
Have you had problems speaking clearly?				0.072
No	32 (89%)	3 (50%)	45 (78%)	
Yes	4 (11%)	3 (50%)	13 (22%)	
Have you had problems with coughing?				0.046
No	29 (81%)	2 (33%)	40 (69%)	
Yes	7 (19%)	4 (67%)	18 (31%)	
Have you had problems going out in public?				0.9
No	33 (92%)	6 (100%)	51 (88%)	
Yes	3 (8.3%)	No (0%)	7 (12%)	

Examining the association between gender and difficulties related to social eating and social contact reported by the respondents. In terms of enjoying meals, 60 (79%) of females and 15 (63%) of males reported no problems, while 16 (21% of females) and nine (38% of males) experienced difficulties. The association between gender and problems enjoying meals was not statistically significant. When it came to eating in front of family and other people, the majority of both females and males reported no issues. However, a higher percentage of females (14, 18%), compared to males (3, 13%), had problems eating in front of others. The associations between gender and eating in front of family and other people were not statistically significant. 

Regarding communication difficulties, 14 (18%) females and 5 (21%) males reported problems talking to other people, and 12 (16%) females and 7 (29%) males experienced difficulties talking on the telephone. However, these associations between gender and communication problems were not statistically significant. Similarly, when asked about speaking clearly and dealing with coughing, there were no statistically significant associations between gender and these issues. 

Lastly, the table examined the association between gender and problems going out in public. Both females and males reported minimal difficulties in this aspect, with no statistically significant association found, as depicted in Table 7.

**Table 7:** Association between gender and trouble with social eating and social contact among the respondents 1: n (%), 2: Pearson's Chi-squared test; Fisher's exact test

	Gender		
Characteristic	Female, N = 76^1^	Male, N = 24^1^	p-value^2^
Have you had problems enjoying your meals?			0.10
No	60 (79%)	15 (63%)	
Yes	16 (21%)	9 (38%)	
Have you had problems eating in front of your family?			0.7
No	69 (91%)	21 (88%)	
Yes	7 (9.2%)	3 (13%)	
Have you had problems eating in front of other people?			0.8
No	62 (82%)	21 (88%)	
Yes	14 (18%)	3 (13%)	
Have you had problems talking to other people?			0.8
No	62 (82%)	19 (79%)	
Yes	14 (18%)	5 (21%)	
Have you had problems talking on the telephone?			0.2
No	64 (84%)	17 (71%)	
Yes	12 (16%)	7 (29%)	
Have you had problems talking in a noisy environment?			>0.9
No	57 (75%)	18 (75%)	
Yes	19 (25%)	6 (25%)	
Have you had problems speaking clearly?			0.080
No	64 (84%)	16 (67%)	
Yes	12 (16%)	8 (33%)	
Have you had problems with coughing?			0.041
No	50 (66%)	21 (88%)	
Yes	26 (34%)	3 (13%)	
Have you had problems going out in public?			0.7
No	69 (91%)	21 (88%)	
Yes	7 (9.2%)	3 (13%)	

When asked about pain in the mouth, 60 (79%) females and 22 (92%) males reported no pain, while 16 (21%) females and 2 (8.3%) males reported experiencing pain. The association between gender and mouth pain was not statistically significant. Similarly, for pain in the jaw, 61 (80%) females and 23 (96%) of males reported no pain, while 15 (20%) females and 1 (4.2%) male reported experiencing jaw pain. The association between gender and jaw pain was not statistically significant. Regarding pain in the throat, 44 (58%) females and 10 (42%) males reported no pain, while 32 (42%) females and 14 (58%) males reported experiencing throat pain. The association between gender and throat pain was not statistically significant. When it came to problems with swallowing, 58 (76%) females and 20 (83%) males reported no difficulties, while 18 (24%) females and 4 (17%) males reported experiencing problems. The association between gender and swallowing difficulties was not statistically significant. The table also explored other difficulties related to swallowing, such as swallowing liquids, pureed food, and solid food. However, the associations between gender and these difficulties were not statistically significant, as depicted in Table 8.

**Table 8:** Association between gender and respondent pain and swallowing difficulties 1: n (%), 2: Fisher's exact test; Pearson's Chi-squared test

	Gender		
Characteristic	Female, N = 76^1^	Male, N = 24^1^	p-value^2^
Have you had pain in your mouth?			0.2
No	60 (79%)	22 (92%)	
Yes	16 (21%)	2 (8.3%)	
Have you had pain in your jaw?			0.11
No	61 (80%)	23 (96%)	
Yes	15 (20%)	1 (4.2%)	
Have you had pain in your throat?			0.2
No	44 (58%)	10 (42%)	
Yes	32 (42%)	14 (58%)	
Have you had problems swallowing?			0.5
No	58 (76%)	20 (83%)	
Yes	18 (24%)	4 (17%)	
Have you had problems swallowing liquids?			0.2
No	58 (76%)	21 (88%)	
Yes	18 (24%)	3 (13%)	
Have you had problems swallowing pureed food?			>0.9
No	60 (79%)	19 (79%)	
Yes	16 (21%)	5 (21%)	
Have you had problems swallowing solid food?			0.13
No	51 (67%)	12 (50%)	
Yes	25 (33%)	12 (50%)	
Have you choked when swallowing?			0.005
No	58 (76%)	24 (100%)	
Yes	18 (24%)	0 (0%)	
Have you had problems opening your mouth wide?			0.5
No	58 (76%)	20 (83%)	
Yes	18 (24%)	4 (17%)	
Have you had problems with your teeth?			0.2
No	37 (49%)	15 (63%)	
Yes	39 (51%)	9 (38%)	
Have you had problems because of losing some teeth?			0.8
No	56 (74%)	17 (71%)	
Yes	20 (26%)	7 (29%)	
Have you had a dry mouth?			0.041
No	50 (66%)	21 (88%)	
Yes	26 (34%)	3 (13%)	
Have you had sticky saliva?			0.4
No	59 (78%)	21 (88%)	
Yes	17 (22%)	3 (13%)	
Have you had swelling in your neck?			0.2
No	0 (0%)	1 (4.2%)	
Yes	53 (70%)	18 (75%)	
Missing	23 (30%)	5 (21%)	
Have you had pain in your shoulder?			0.4
No	53 (70%)	19 (79%)	
Yes	23 (30%)	5 (21%)	
Have you had problems raising your arm or moving it sideways?			0.4
No	57 (75%)	20 (83%)	
Yes	19 (25%)	4 (17%)	
Had you felt numbness in your hands or feet’s?			0.13
No	44 (58%)	18 (75%)	
Yes	32 (42%)	6 (25%)	

## Discussion

Quality of life (QoL) assessment includes physical, mental, social, and psychological functioning. Most of our participants were females (76%) with a good level of education (bachelor's = 30%), and 64% were not working at the time of the study. More than half underwent total thyroidectomy (58%), 36% underwent hemithyroidectomy, and only 6.0% underwent subtotal thyroidectomy operations among the study participants. There was no significant difference between males and females regarding elements of trouble with social eating and social contact, except for the finding that females experience more problems with coughing (p-value =0.041). Nevertheless, there was no significant difference in the assessment of pain and swallowing difficulties according to gender, except for the items: being choked when swallowing (p-value =0.005) and having a dry mouth (p-value =0.041). 

When assessing sense problems, body satisfaction, and sexual satisfaction among the study respondents, the most-rated items were worry about health in the future (63%) and feeling worried because of test results (57%). Similar findings were reported by another study in Saudi Arabia: worry about the test results (mean = 2.37) and worry about future health (mean = 2.36). Nevertheless, symptoms like numbness in hands or feet were reported by 38% of our participants, compared to a mean score of 2.32 ± 1.22. However, the items “problems with your teeth" (48%) and “problems with swallowing solid food" (37%) were more perceived symptoms by our participants, yet shoulder pain (2.04 ± 1.18) and skin problems (2.04 ± 1.18) were more appreciated symptoms by Saudi participants from the Riyadh area [7]. 

A study conducted in Canada found no statistically significant difference in the global quality of life between the total and hemithyroidectomy groups. Yet, thyroid carcinoma survivors who underwent hemithyroidectomy have more to worry about cancer recurrence when compared with the total thyroidectomy group (p= .02) [15]. 

This study found no statistically significant difference in quality of life among participants who underwent total, subtotal, or hemithyroidectomy in terms of sense problems, body and sexual satisfaction, and social aspects. Similar findings were reported by Alyousef et al., where the difference between patients' symptoms according to type of surgery didn’t approach statistical significance (p = 0.593) [7]. Nevertheless, a cohort study conducted in China investigating health-related quality of life (HrQoL) among differentiated thyroid carcinoma patients concluded that, in the first three months, HrQoL among the total thyroidectomy group is worse with more postoperative problems, yet this difference disappears in the long term (6, 12 months) [16]. A review article raised a different finding; it reports that, when compared with hemithyroidectomy patients, total thyroidectomy patients may experience more impairment in social and physical HrQoL, yet there is no statistical difference in psychological HrQoL or long-term global HrQoL [17]. However, another interesting prospective is the comparison between QoL according to surgical approach, endoscopic versus classical open procedure. The study concluded that endoscopic thyroidectomy patients showed better improvement in physical and emotional functions compared to open thyroidectomy. Nevertheless, postoperative pain was more commonly reported with the endoscopic approach of thyroidectomy [18].

Based on the outcome of this study, it is recommended that patients be actively involved in decision-making and be thoroughly informed about complications both pre- and post-operatively. They should also be involved in managing and increasing their quality of life after thyroidectomy procedures of any type. 

Strengths and limitations 

The limitations of this study include the use of medical records, which could be inaccurate in reporting, and the assessment in the long term, which raised the concern of recall bias, yet these limitations were accounted for by the relatively large sample size to minimize the effect of such bias.

## Conclusions

This study concluded that the most reported problems post-thyroidectomy in the long term were worry about health in the future and worry about test results, which necessitate paying more attention to these aspects and discussing them with thyroid cancer survivors both preoperatively and in the postoperative follow-up with the help of a social worker. Nevertheless, there is no significant difference in life quality between total thyroidectomy and hemithyroidectomy in terms of quality of life. Further future research with a larger sample size and more centers involved is recommended.
